# Fast food consumption among 12- to 17-year-olds in Germany – Results of EsKiMo II

**DOI:** 10.25646/6398

**Published:** 2020-03-04

**Authors:** Ramona Moosburger, Clarissa Lage Barbosa, Marjolein Haftenberger, Anna-Kristin Brettschneider, Franziska Lehmann, Anja Kroke, Gert B. M. Mensink

**Affiliations:** 1 Robert Koch Institute, Berlin Department of Epidemiology and Health Monitoring; 2 Formerly Robert Koch Institute, Berlin Department of Epidemiology and Health Monitoring; 3 Fulda University, Department of Nutritional, Food and Consumer Sciences

**Keywords:** FAST FOOD CONSUMPTION, ADOLESCENTS, GERMANY, NUTRITION SURVEY, ESKIMO II, HEALTH MONITORING

## Abstract

Consuming high amounts of fast food can lead to an excessive intake of energy and subsequently promote obesity. Obesity increases a person’s risk for diabetes and cardiovascular diseases. The second wave of the German Health Interview and Examination Survey for Children and Adolescents (KiGGS Wave 2, 2014–2017) included the Eating study as a KiGGS Module (EsKiMo II, 2015–2017) which assessed the self-reported dietary habits of children and adolescents in Germany. The analysis of the data permits an overview of the fast food consumption of 12- to 17-year-olds (n=1,353). Girls consume 57.5 grams and boys 86.3 grams of fast food per day on average (around 400 grams and 600 grams per week, respectively). Pizza is the most consumed fast food product, followed by filled pita and sausage/meat products such as curry sausage. Adolescent girls on average get 6.5% and boys 7.8% of total daily energy intake from fast food. 23% of 12- to 17-year-olds get at least 10% of their daily energy intake from fast food (high consumers). Significant differences between the proportion of high consumers exist regarding sex, age, socioeconomic status, community size, type of school and media consumption. Compared to EsKiMo I (2006), girls’ daily energy intake from fast food has remained nearly constant, whereas that of boys has dropped substantially. From a nutrition physiology perspective, the aim should be to further reduce fast food consumption.

## 1. Introduction

The environment of many children and adolescents’ today is conducive to overweight and obesity [1]. Results from the second wave of the German Health Interview and Examination Survey for Children and Adolescents (KiGGS Wave 2, 2014–2017) have shown that 15.4% of 3- to 17-year-old children and adolescents in Germany are overweight and/or obese and 5.9% obese [2]. Being obese at childhood and adolescent age is considered to be a strong predictor for obesity at adult age. Obesity is often related to stigmatisation and health issues and is also an important risk factor for non-communicable diseases such as cardiovascular diseases and type 2 diabetes [1, 3].

Becoming overweight or obese depends not only on physical activity, an important factor is also dietary behaviour, with a number of factors influencing food choices. These include taste and individual preferences, but also price, availability and changes to daily routines which may lead a person to eat ready made or takeaway meals more frequently [4]. The continuously growing turnover and opening of new fast food restaurants by the major chains also in Germany is proof of fast food’s great popularity [5–7].

**KiGGS Wave 2**

Second follow-up to the German Health Interview and Examination Survey for Children and Adolescents

**Data owner:** Robert Koch Institute

**Aim:** Providing reliable information on health status, health-related behaviour, living conditions, protective and risk factors, and health care among children, adolescents and young adults living in Germany, with the possibility of trend and longitudinal analyses

**Study design**: Combined cross-sectional and cohort study

**Cross-sectional study in KiGGS Wave 2**

**Age range:** 0–17 years

**Population:** Children and adolescents with permanent residence in Germany

**Sampling:** Samples from official residency registries - randomly selected children and adolescents from the 167 cities and municipalities covered by the KiGGS baseline study

**Sample size:** 15,023 participants

**KiGGS cohort study in KiGGS Wave 2**

**Age range:** 10–31 years

**Sampling:** Re-invitation of everyone who took part in the KiGGS baseline study and who was willing to participate in a follow-up

**Sample size:** 10,853 participants

**KiGGS survey waves**

▶ KiGGS baseline study (2003–2006), examination and interview survey
▶ KiGGS Wave 1 (2009–2012), interview survey
▶ KiGGS Wave 2 (2014–2017), examination and interview survey

More information is available at www.kiggs-studie.de/english

In general, fast food is highly processed and highly standardized to ensure preparation within a very short time (in fast food restaurants and at snack stands) and eaten immediately, often on-the-way [8]. Fast food is mostly eaten as a snack and usually contains less essential nutrients and dietary fibre. However, the glycaemic index (measure describing the effects of foods containing carbohydrates on blood glucose levels) of fast food is often higher than that of a full meal. This means fast food is, in spite of large portion sizes, less satiating, which therefore can lead to higher total daily energy intake [9, 10]. Adolescents often feel a desire to distinguish themselves from the food habits of their parents, which in combination with the unconventional atmosphere of fast food that is often eaten without cutlery and plates [11] could be a reason why they often opt for fast food [12].

Due to its high degree of processing and its low micronutrient density, fast food is viewed critically for its role in the development of overweight and obesity [13]. Fast food is usually rich in fat, contains high amounts of highly processed carbohydrates, and is rich in salt and hidden sugars [8, 14], and therefore considered unhealthy [11]. Nutrition-physiologically healthy fast food does exist, yet only plays a marginal role [8]. Studies have shown that adolescents who live in areas with a greater availability of fast food also ate more fast food, and their overall dietary patterns were also less healthy [15]. Consuming large amounts of fast food is moreover related to a higher risk to develop diabetes, metabolic syndrome and cardiovascular diseases [14]. Numerous studies have indicated a link between regularly consuming fast food and gaining weight [4, 16, 17]. As a measure to prevent overweight, obesity and non-communicable diseases and in the interest of a balanced diet, fast food should not be consumed regularly [18, 19].

To survey the dietary habits of children and adolescents in Germany, KiGGS Wave 2 included the Eating study as a KiGGS Module (EsKiMo II, 2015–2017) based on self-reported data as one of its modules. Data analysis provides findings on the fast food consumption habits of 12- to 17-year-olds. This could help develop recommendations for prevention. Based on EsKiMo II data, the aim of this analysis is to describe fast food consumption of 12- to- 17-year-olds in Germany and how fast food contributes to daily energy intake against the backdrop of sociodemographic and lifestyle factors. Furthermore, the proportion of 12- to 17-year-olds was determined, who get at least ten percent of their total energy from fast food (high consumers).

## 2. Methodology

### 2.1 Study design and study population

EsKiMo II is a survey of the dietary habits of 6- to 17-year-old children and adolescents in Germany. The study was conducted as a module of KiGGS Wave 2 between June 2015 and September 2017. KiGGS forms part of the health monitoring system at the Robert Koch Institute (RKI) and includes repeated cross-sectional surveys of children and adolescents aged 0 to 17 that are representative for Germany. The KiGGS baseline study (2003–2006) was conducted as an examination and interview survey, the first follow-up study (KiGGS Wave 1, 2009–2012) as a telephone-based interview survey and KiGGS Wave 2 (2014–2017) as an examination and interview survey. Participants were randomly selected from the population registries of 167 cities and municipalities representative for Germany which had already been selected for the baseline study. The concept and design of KiGGS have been described in detail elsewhere [20, 21]. Overall 15,023 children and adolescents (7,538 girls, 7,485 boys) took part in KiGGS Wave 2 (response rate 40.1%). 3,567 children and adolescents (1,801 girls, 1,766 boys) took part in the examinations (response rate 41.5%) [20]. EsKiMo II was conducted with a subsample of the KiGGS Wave 2 cross sectional survey. 2,644 participants (1,361 girls, 1,283 boys) took part in EsKiMo II [22]. The current analysis looked at the dietary data of 12- to 17-year-olds (n=1,353; 727 girls, 626 boys). The study design and procedures of EsKiMo II have been described in detail elsewhere [22–24].

**EsKiMo II**

Second Wave of the Eating study as a KiGGS Module, 2015–2017

**Acronym: EsKiMo – E**ating **s**tudy as a **Ki**GGS **Mo**dule

**Implementation:** Robert Koch Institute

**Aim:** Providing an up-to-date representative overview of the dietary habits of children and adolescents aged 6 to 17 in Germany.

**Study design:** Cross-sectional study based on a modified diet history interview and food records

**Population:** Children and adolescents with permanent residence in Germany

**Sampling:** EsKiMo II participants are randomly selected from the cross-sectional sample of KiGGS Wave 2 (registry office sample). Being invited to EsKiMo II requires participation in KiGGS Wave 2.

**Age range:** 6 to 17 years

**Sample size:** 2,644 participants

**Survey period:** June 2015 – September 2017

More information in German is available at www.rki.de/eskimo

### 2.2 Indicators

#### Fast food consumption

The 12- to 17-year-old participants were asked about the food they had eaten during the last four weeks using a computer-based dietary history interview (Dietary Interview Software for Health Examination Studies, DISHES). DISHES is a survey instrument validated for adults [25]. Trained nutritionists conducted the dietary interview during home visits. Consumption frequencies and portion sizes were enquired for all foods and each meal. To better evaluate portion sizes, model plates and a photo book [26, 27] were used. The data was coded based on version 3.02 of the German Food Code and Nutrient Data Base (BLS) [28].

The assessed foods that were categorized as fast food were divided into eight categories (Table 1). This selection was based on the same definition of fast food as in EsKiMo I and used food names, because data on the place of consumption was not collected and consequently not taken into account (for example for pizza). Sauces, which are frequently consumed together with fast food and often contain a lot of fat and/or sugar, are included here as fast food too.

Fast food intake in grams and in kilocalories (kcal) was calculated per person per day as well as total daily energy intake. The proportion of energy from fast food was calculated by dividing the total energy intake from fast food by total energy intake. Like in EsKiMo I those who got at least ten percent of their daily energy from fast food were defined as high consumers [29]. Other institutions also recommend limiting the energy intake from specific unhealthy foods to ten percent of total energy intake [30].

#### Sociodemographic and lifestyle factors

The analysis included information on sociodemographic and lifestyle factors assessed within KiGGS Wave 2 for the 12- to 17-year-olds. Family socioeconomic status (SES) was defined based on a multi-dimensional index which considers the answers parents provided in questionnaires on school education and training, professional status and equivalised household income. SES allows the definition of a low, medium and high status group [31]. Participants were considered as having a migration background if they themselves had migrated, or, if at least one of their parents had not been born in Germany, or, if both parents had migrated or did not hold German citizenship [32]. The municipalities in which participants lived were divided into four groups based on community sizes (as of 31 December 2015) (<5,000, 5,000–< 20,000, 20,000–<100,000 and ≥100,000 inhabitants) [33]. Schools were classified as secondary, secondary modern, comprehensive, grammar (Hauptschule, Realschule, Gesamtschule or Gymnasium) and other schools. For the analysis, Germany was divided into five regions: North West (Schleswig-Holstein, Bremen, Hamburg, Lower Saxony), North Rhine-Westphalia, Center (Hesse, Rhine-land-Palatinate, Saarland), East (Berlin, Brandenburg, Mecklenburg Western Pomerania, Saxony, Saxony Anhalt, Thuringia) and South (Bavaria, Baden-Wuerttemberg). Data on media usage was divided into three categories based on time spent watching TV/DVDs, playing game consoles/computer games and other PC/internet activities: less than three hours, three to six hours and over six hours per day. Self-reported data on sport activities was summarised into four groups: no sport, less than two hours, two to four hours and over four hours per week.

### 2.3 Statistical methods

Mean values and 95% confidence intervals for daily fast food consumption and the proportion of energy from fast food stratified by sociodemographic and lifestyle factors were determined for girls and boys. Due to the unequal distribution of fast food consumption levels, relevant distribution factors (median and interquartile ranges) were described. Multivariate variance analysis was applied to determine the relationship between the determined percentage of energy from fast food and sociodemographic and lifestyle factors. Differences depending on sociodemographic and lifestyle factors were tested using f-tests. The proportion of high consumers was stratified with 95% confidence intervals by sociodemographic and lifestyle factors. Differences in the proportion of high consumers were tested for significance based on chi-squared tests. A statistically significant difference between groups is assumed when the corresponding p-value is smaller than 0.05.

Missing data led to the exclusion of a number of participants from individual indicators: 19 from the socioeconomic status; 9 from the migration background; 49 from the type of school; 64 from sports activities and 41 from the media consumption indicators, respectively.

The calculations were carried out using an adapted weighting factor for EsKiMo II that corrects deviations within the sample from the population structure with regard to regional structure (rural area/urban area), age (in years), sex, federal state (as of 31 December 2015), German citizenship (as of 31 December 2014) and parent level of education according to the CASMIN classification (Comparative Analysis of Social Mobility in Industrial Nations [34], Microcensus 2013 [35]), as well as differences in participation in the nutrition survey regarding seasonality, family SES and type of school. To account for the cluster design of the sample in the calculation of confidence intervals and p-values, the analyses were conducted with the survey procedures of the SAS^®^ statistics software version 9.4 (SAS Institute, Cary, NC, USA).

## 3. Results

**Figure 1:**
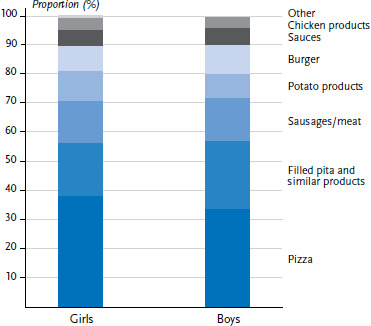
Proportion of types of fast food in percentage of total daily fast food consumption (grams per day) by sex Source: EsKiMo II (2015–2017)

Nearly all 12- to 17-year-olds (97.8%) reported that they had eaten fast food at least once during the four weeks before the interview. On average, they consume 72.3 grams of fast food (158 kcal) per day. 11.5% of participants (7.3% of girls and 15.5% of boys) consume more than 150 grams of fast food per day. Girls on average eat 57.5 grams (125 kcal) and boys 86.3 grams of fast food (188 kcal) per day (data not shown). For both sexes, pizza is proportionally the most consumed fast food, followed by filled pita, as well as sausage/meat products (Figure 1).

12- to 17-year-olds on average get 7.2% of their daily energy from fast food (data not shown). Compared to boys (7.8%), girls (6.5%) get a smaller proportion of their daily energy from fast food. The proportion of energy from fast food increases with age and is lower for those with higher family SES. Girls and boys with a migration background get a higher proportion of their energy from fast food than boys and girls without a migration background. Compared to their peers from other regions, boys and girls in the eastern states of Germany get the lowest percentage of their energy from fast food. Pupils at secondary, secondary modern and comprehensive schools get a larger proportion of their energy from fast food than those at grammar schools. Adolescents who spend more time with electronic media, get a larger proportion of their energy from fast food than their peers with lower levels of media usage. Community size and levels of sport activity had no influence on fast food consumption neither in girls nor boys (Table 2).

###  

#### Multivariate analyses

**Figure 2:**
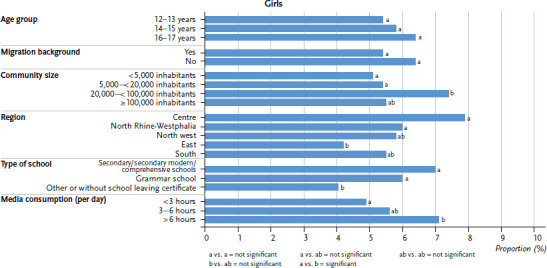

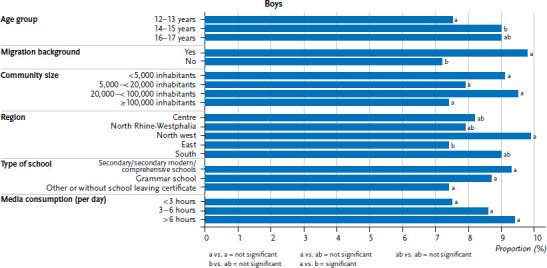
Average proportion of energy from fast food after multivariate adjustment by sex (n=727 girls, n=626 boys) Source: EsKiMo II (2015–2017)

In the following step, all variables that potentially have an influence were included within a statistical model. When simultaneously adjusted for all independent variables, 12- to 17-year-old girls’ average daily energy intake from fast food in Germany is significantly associated to type of school, community size, region and amount of time spent with media per day. Pupils of secondary, secondary modern, comprehensive and grammar schools get a significantly higher proportion of their daily energy from fast food than pupils from other school types. Girls in communities with 20,000–<100,000 inhabitants get a significantly higher amount of their energy from fast food than girls in communities with a population of under 5,000 or of 5,000–< 20,000. Girls from North Rhine-Westphalia and the central region of Germany get a significantly higher percentage of their energy from fast food than those in the eastern states of Germany. Girls who spend over six hours per day on media get a significantly higher amount of their daily energy from fast food than girls who spend less than three hours on media (Figure 2).

When adjusted for all independent variables, the average daily percentage of energy 12- to 17-year-old boys get from fast food is significantly associated to age group, migration background and the region, in which they live. 14- to 15-year-olds get a significantly higher percentage of their daily energy from fast food than 12- to 13-year-olds. Boys with a migration background get a significantly higher percentage of their energy from fast food than those with no migration background. Furthermore, boys from the eastern states of Germany get a significantly lower percentage of their energy from fast food than boys from north western Germany (Figure 2). There are no significant differences in percentage of energy from fast food by family SES or levels of sport activity of girls and boys.

#### Proportion of high consumers of fast food

Table 3 shows the proportion of high consumers who get at least ten percent of their daily energy from fast food. This applies to around 23.0% of 12- to 17-year-olds. The proportion of high consumers is significantly higher for boys (26.5%) than girls (19.3%). The proportion of high consumers increases significantly with age. Boys and girls with low SES (35.5%) get at least ten percent of their daily energy from fast food significantly more often than boys and girls with high SES (17.4%). A significantly higher number of girls and boys get at least ten percent of their daily energy from fast food in communities with a population between 20,000 and 100,000 people than in communities with between 5,000 and 20,000 people. In the eastern states of Germany, the percentage of high consumers (15.9%) is lower, and in central Germany it is higher (30.5%) compared to other regions. However, none of these regional differences are significant. The proportion of high consumers among those who go to secondary, secondary modern and comprehensive schools is significantly higher than for those who attend grammar schools. 32.9% of participants who spend a lot of time on media, get at least ten percent of their daily energy from fast food, whereas this applies to only 14.7% of those with low levels of media consumption. Migration background and levels of sport activity had no significant impact on high consumer figures.

## 4. Discussion

During a four-week period, nearly all 12- to 17-year-olds had consumed fast food at least once. Almost one quarter of boys and girls get at least ten percent of their daily energy from fast food. Based on the same definition of fast food, EsKiMo I (2006) had found that adolescents get 191 kilocalories (kcal) per day from fast food (girls 126 kcal, boys 252 kcal) [11]. EsKiMo II data shows that while this average value has remained the same for girls during the last ten years, it has dropped for boys (girls 125 kcal, boys 188 kcal). Reasons for the changes regarding fast food consumption were not recorded in EsKiMo.

When putting these results into perspective, it is important to consider the possibly limited comparability with other studies. One reason is the lack of a uniform definition of fast food. Fast food is a highly culture-dependent term and subject to changes over time.

EsKiMo II results show that boys get a significantly higher percentage of their average energy from fast food and more often at least ten percent of their energy from fast food than girls. This indicates differences in the dietary habits of girls and boys. One reason could be the less restrained eating habits of boys [36]. Some studies have shown significant age differences in the fast food consumption of children and adolescents. Older children, thereby, on average eat more fast food than younger children [37–39]. A Canadian study, covering the entire over-two-year old population, found that the group with the highest levels of fast food consumption (248 kcal per day) was male adolescents (14- to 18-year-olds) [12]. The results presented here likewise confirm significant age differences for levels of fast food consumption among 12- to 17-year-old boys. Furthermore, compared to the youngest, the proportion of high consumers is significantly higher among the older participants. Adolescents seek autonomy and independence and spend more time outside of the home during their leisure time. The significance of belonging to a peer group becomes very important during this age phase. Fast food is very popular among adolescents and is considered a part of a diet that expresses a lifestyle [40].

The greater spread of fast food consumption among 12- to 17-year olds with low SES is a reason that there are no significant differences by SES of average fast food consumption (Table 2). However, girls and boys with low SES get at least ten percent of their daily energy from fast food significantly more often than girls and boys with high SES.

Very likely, the significant difference in the average proportion of energy boys with and without migration background get from fast food is related to the definition used here. Our definition includes food that culturally belongs to the regions many migrants come from and which are therefore presumably consumed more frequently by this group. Notwithstanding, analyses from a number of countries indicate a higher density of fast food restaurants in neighbourhoods with a higher percentage of migrants [41].

The percentage of energy boys and girls get from fast food is highest for communities with a population between 20,000 and 100,000 people. In these communities, the proportion of 12- to 17-year-olds who get at least ten percent of their daily energy from fast food is also highest compared to communities of other sizes. A potential explanation, why girls and boys in the eastern states of Germany eat the least fast food is possibly the lower density of fast food restaurants in rural areas [41], and the east has more rural areas than other parts of Germany. Furthermore, in the former East, school cantines are more common than in the former West German states and are frequented by more children [42]. Adolescents who regularly eat at school very likely eat fast food less frequently.

The connection between media consumption and choosing more unhealthy foods has repeatedly been observed for adolescents [43–45]. The effect has also been related to TV advertising for unhealthy foods [46, 47]. However, based on the structure of the data, it was not possible to establish a cause-and-effect relationship between fast food and media consumption.

One limitation of the study is that the data does not allow determining where and in which combination participants ate. Precisely recording where foods were eaten or bought and in which combination participants consume them is however hardly possible for a four-week period. Furthermore, from a nutrition physiology perspective, it is more important, what participants eat and not where. From this angle we can analyse fast food independently of where it is consumed. However, meals classified as fast food that are prepared at home can, nutrition physiologically, be more valuable than those bought outside.

A further limitation is the study’s cross-sectional design. Cross-sectional data can only reveal relationships, it is not possible to derive causalities. Self-reported data on dietary habits can also be biased due to what people feel are socially expected answers [48]. Frequently, people do under-report the consumption of foods that society considers not favourable such as fast food. Inversely, foods considered as healthy could well be over-reported, and then reduce the relative amounts of energy participants get from fast food. A further source of bias could be that participants do not correctly remember what they have eaten. To minimise the effects of such mistakes, the DISHES software, which includes plausibility tests, highly standardises the collection of data on food consumption. Furthermore, measures of quality assurance such as training interviewers and the presence of field coordinators during the field phase were conducted to ensure that interviews were performed properly [22]. Moreover, it is important to bear in mind that fast food was centrally defined for the analysis and not by the participants. The definition can therefore be considered as standardised and transparent.

A key strength of the study is that, based on a broad sample, EsKiMo II provides representative data on the dietary habits of children and adolescents from across Germany. Through the connection of this study to KiGGS Wave 2, relationships with a number of sociodemographic and lifestyle factors can be analysed. The 40.1% response rate of KiGGS Wave 2 is satisfactory and comparable to the reponse rates achieved by other health monitoring studies [20]. The re-participation rate of EsKiMo II of 59.4% was thereby clearly higher [22]. To further optimise representativity, weighting factors were developed and applied in the analyses.

###  

#### Conclusion and Outlook

Nearly one quarter of 12- to 17-year-olds gets at least ten percent of their daily energy from fast food. During the last ten years, levels of fast food consumption have remained more or less equal for girls, but dropped for boys. From a health policy and prevention perspective, reducing levels of fast food consumption remains important. One possible approach could be the provision of healthier fast food options and thereby increase the appeal of balanced and health promoting diets. Besides, it has been discussed that the number of fast food restaurants and snack stands in proximity to schools could be reduced [50], similar to the approach taken with cigarette machines [49], while making school meals healthier and more attractive. It could also be worth considering introducing nutrition as a school subject. Some countries have already introduced policies to reduce fast food consumption [50–52]. These include a tax on unhealthy foods and a simultaneous reduction of tax on, for instance, fruit and vegetables, to stimulate changes in consumption patterns. A 20% increase in the price of fast food would presumably lead to a ten percent cut in consumption [51]. A systematic review among adolescents in the US showed that increasing the cost of fast food can lead to weight reduction among adolescents. Accordingly, adolescents with low or medium SES and a higher body mass index would benefit most from a tax on fast food [51]. Further research is required to understand the relationship between consuming fast food and other types of food. A possible approach could be to analyse how much sweets, salty snacks and soft drinks, fast food high consumers in Germany eat.

## Key statements

- In terms of quantity, pizza is the most consumed fast food among 12- to 17-year-olds.
- Girls consume on average around 400 grams of fast food per week, boys 600 grams.
- Girls on average get 6.5% and boys 7.8% of their daily energy from fast food.
- 23.0% of 12- to 17-year-olds get at least ten percent of their daily energy from fast food.
- Fast food consumption among girls and boys remains relatively high.

## Figures and Tables

**Table 1:** Types of fast food and selected foods Source: Own table

Fast food category	Selected foods
Pizza	All kinds of pizza
Burger	Hamburgers, cheeseburgers, gyrosburgers, chicken burgers, veggie burgers, fish burgers
Filled pita bread and similar products	Döner kebab, falafel, lahmacun, gyros, börek, pide, wrap
Sausages/Meat	Curry sausage, sausage (also chicken and soy sausages), hot dog, meat loaf on roles, meatballs in bread
Potatoe products	French fries, country potatoes
Chicken products	Roast chicken, chicken nuggets, chicken wings
Sauces	Fast food chain sauces, mayonnaise, remoulade, ketchup
Other foods	Spring roles, mozzarella sticks, fried fish in bread, matjes roles, onion rings, vegetarian nuggets, fried squid rings

**Table 2:** Daily percentage of energy from fast food by sex, sociodemographic and lifestyle factors (n=727 girls, n=626 boys)^*^ Source: EsKiMo II (2015–2017)

	Girls						Boys					
Variable	n	Mean (%)	(95% CI)	Median (%)	1^st^ quartile (%)	3^rd^ quartile (%)	n	Mean (%)	(95% CI)	Median (%)	1^st^ quartile (%)	3^rd^ quartile (%)
**Total**	**727**	**6.5**	**(5.9-7.2)**	**5.3**	**2.7**	**8.9**	**626**	**7.8**	**(7.1-8.5)**	**6.4**	**3.5**	**10.3**
**Age group**												
12–13 years	248	5.5	(4.8–6.2)	4.6	2.2	7.5	250	7.0	(6.2–7.8)	5.8	3.6	9.3
14–15 years	259	6.7	(5.5–7.8)	5.1	2.7	8.8	204	8.3	(7.3–9.3)	7.6	3.7	10.6
16–17 years	220	7.4	(6.2–8.6)	5.7	3.4	10.6	172	8.2	(6.7–9.7)	6.0	3.0	12.3
**Socioeconomic status**												
Low	76	7.6	(5.7–9.5)	5.6	2.4	12.0	53	9.1	(6.1–12.1)	6.8	3.6	12.4
Medium	473	6.5	(5.8–7.2)	5.4	3.0	9.0	390	7.8	(7.1–8.5)	6.6	3.6	10.0
High	167	5.5	(4.5–6.4)	4.3	2.3	7.2	175	7.1	(6.1–8.2)	6.1	3.4	9.0
**Migration background**												
Yes	88	7.0	(5.3–8.6)	5.4	2.3	11.8	59	10.0	(7.2–12.7)	7.7	4.3	10.5
No	631	6.5	(5.9–7.2)	5.3	3.0	8.7	566	7.4	(6.8–8.0)	6.2	3.4	10.1
**Community size**												
<5,000 inhabitants	165	6.2	(5.3–7.1)	5.2	3.4	7.8	154	7.7	(6.6–8.9)	6.4	3.3	9.8
5,000–<20,000 inhabitants	198	5.9	(5.2–6.7)	5.4	2.6	8.6	174	7.1	(6.1–8.1)	5.9	3.1	9.9
20,000–<100,000 inhabitants	199	7.9	(6.3–9.5)	5.4	3.0	11.8	182	8.9	(7.1–10.6)	6.6	4.0	12.2
≥100,000 inhabitants	165	6.0	(4.8–7.1)	4.8	2.0	8.0	116	7.6	(6.3–8.8)	6.8	3.7	9.9
**Region**												
North west	86	6.3	(4.9–7.7)	4.8	1.9	9.1	83	9.1	(6.9–11.3)	7.1	3.8	12.5
North Rhine-Westphalia	139	7.5	(5.9–9.2)	5.8	2.8	10.2	113	7.6	(6.5–8.8)	7.3	4.1	9.9
Centre	83	8.1	(5.9–10.2)	6.6	3.1	11.2	79	7.9	(5.4–10.4)	5.9	2.4	10.6
East	249	5.0	(4.4–5.7)	3.9	1.8	6.8	216	6.2	(5.2–7.3)	4.9	2.4	8.0
South	170	6.1	(5.2–6.9)	5.0	3.3	7.7	135	8.1	(7.0–9.3)	6.7	4.1	10.1
**Type of school**												
Secondary/secondary modern/comprehensive schools	272	7.5	(6.5–8.5)	5.8	2.8	10.3	260	8.0	(6.8–9.2)	7.1	3.9	11.4
Grammar school	392	5.9	(5.1–6.7)	4.7	2.4	7.7	300	7.4	(6.6–8.2)	6.2	3.3	10.1
Other type of school, without school leaving certificate	34	5.8	(4.8–6.8)	5.6	4.2	7.1	46	5.9	(3.5–8.2)	5.8	3.4	7.5
**Sport activity (per week)**												
None	175	7.3	(6.1–8.6)	5.8	3.3	11.7	92	7.4	(6.0–8.8)	6.3	3.5	9.9
<2 hours	152	5.8	(4.8–6.8)	4.8	2.5	8.5	85	8.6	(6.8–10.5)	7.7	3.5	12.5
2–4 hours	200	7.0	(5.5–8.5)	5.2	2.8	9.0	144	8.1	(6.2–9.9)	6.3	3.0	10.1
>4 hours	168	5.9	(5.1–6.8)	5.4	2.3	8.8	273	7.8	(6.8–8.8)	6.6	3.6	10.0
**Media consumption (per day)**												
<3 hours	243	5.4	(4.6–6.2)	4.4	2.3	7.2	198	6.6	(5.9–7.3)	5.7	3.5	8.8
3–6 hours	271	6.4	(5.4–7.4)	5.6	2.3	9.1	265	8.0	(6.9–9.2)	6.6	3.4	10.9
>6 hours	188	8.1	(6.8–9.5)	6.8	3.8	11.4	147	9.0	(7.4–10.6)	6.8	3.9	13.1

CI = confidence interval, n = unweighted number of participants

^*^Missing data led to the exclusion of a varying number of participants from the analysis for individual indicators.

**Table 3:** Proportion of 12- to 17-year-olds, who get at least ten percent of their daily energy from fast food by sociodemographic and lifestyle factors (n=727 girls, n=626 boys)^*^ Source: EsKiMo II (2015–2017)

Variable	n	%	(95% CI)	p-value
**Sex**				
Girls	727	19.3	(14.3–24.3)	0.0392
Boys	626	26.5	(21.6–31.4)	
**Age group**				
12–13 years	498	17.3	(12.9–21.7)	0.0144
14–15 years	463	22.9	(17.1–28.7)	
16–17 years	392	28.3	(21.9–34.7)	
**Socioeconomic status**				
Low	129	35.5	(22.8–48.1)	0.0030
Medium	863	21.1	(17.5–24.7)	
High	342	17.4	(12.1–22.7)	
**Migration background**				
Yes	147	29.9	(19.6–40.3)	0.0857
No	1,197	21.6	(18.1–25.1)	
**Community size**				
<5,000 inhabitants	319	20.1	(14.3–25.9)	0.0181
5,000–<20,000 inhabitants	372	17.8	(12.6–22.9)	
20,000–<100,000 inhabitants	381	31.2	(23.2–39.2)	
≥100,000 inhabitants	281	21.8	(13.7–29.9)	
**Region**				
North west	169	28.9	(19.3–38.4)	0.0624
North Rhine-Westphalia	252	24.6	(16.6–32.6)	
Centre	162	30.5	(15.3–45.8)	
East	465	15.9	(11.7–20.0)	
South	305	19.1	(13.3–24.8)	
**Type of school**				
Secondary/secondary modern/comprehensive schools	532	27.9	(21.9–34.0)	0.0374
Grammar schools	692	20.5	(16.1–24.8)	
**Sport aktivity (per week)**				
None	267	25.6	(16.8–34.4)	0.8404
<2 hours	237	24.9	(16.3–33.5)	
2–4 hours	344	22.8	(16.4–29.2)	
>4 hours	441	21.8	(16.6–27.0)	
**Media consumption (per day)**				
<3 hours	441	14.7	(10.0–19.3)	0.0004
3–6 hours	536	23.0	(17.0–28.9)	
>6 hours	335	32.9	(25.3–40.5)	

CI = confidence interval, n = unweighted number of participants

^*^Missing data led to the exclusion of a varying number of participants from the analysis for individual indicators.
